# Erythroid Progenitor Cells Expanded from Peripheral Blood without Mobilization or Preselection: Molecular Characteristics and Functional Competence

**DOI:** 10.1371/journal.pone.0009496

**Published:** 2010-03-02

**Authors:** Claudia Filippone, Rauli Franssila, Arun Kumar, Leena Saikko, Panu E. Kovanen, Maria Söderlund-Venermo, Klaus Hedman

**Affiliations:** 1 Department of Virology, Haartman Institute, University of Helsinki and Helsinki University Laboratory Division, Helsinki, Finland; 2 Department of Pathology, Haartman Institute, University of Helsinki, Helsinki, Finland; University of Kansas Medical Center, United States of America

## Abstract

**Background:**

Continued development of in-vitro procedures for expansion and differentiation of erythroid progenitor cells (EPC) is essential not only in hematology and stem cell research but also virology, in light of the strict erythrotropism of the clinically important human parvovirus B19.

**Methodology/Principal Findings:**

We cultured EPC directly from ordinary blood samples, without *ex vivo* stem cell mobilization or CD34+ cell *in vitro* preselection. Profound increase in the absolute cell number and clustering activity were observed during culture. The cells obtained expressed the EPC marker combination CD36, CD71 and glycophorin, but none of the lymphocyte, monocyte or NK markers. The functionality of the generated EPC was examined by an *in vitro* infection assay with human parvovirus B19, tropic for BFU-E and CFU-E cells. Following infection (i) viral DNA replication and mRNA production were confirmed by quantitative PCR, and (ii) structural and nonstructural proteins were expressed in >50% of the cells. As the overall cell number increased 100–200 fold, and the proportion of competent EPC (CD34+ to CD36+) rose from <0.5% to >50%, the *in vitro* culture procedure generated the EPC at an efficiency of >10 000-fold. Comparative culturing of unselected PBMC and ex vivo-preselected CD34+ cells produced qualitatively and quantitatively similar yields of EPC.

**Conclusions/Significance:**

This approach yielding EPC directly from unmanipulated peripheral blood is gratifyingly robust and will facilitate the study of myeloid infectious agents such as the B19 virus, as well as the examination of erythropoiesis and its cellular and molecular mechanisms.

## Introduction

The basic mechanisms of stem cell proliferation and differentiation leading to erythropoiesis are well established. In vitro studies on this topic have been carried out with progenitor cells obtained not only from bone marrow, but also from foetal liver and peripheral blood [1]–[6]. The erythropoietic growth factors affect the progenitors in all these locations [3], and many procedures have been undertaken to reproduce the erythroid maturation *in vitro* including initial selection of the CD34+ cells [7]–[11], adherence depletion [1], [3], [12], [13] and phased culturing [6], [12], [14]. *In vitro* culture of selected CD34+ cells following G-CSF mobilization of peripheral blood stem cells (PBSC) was recently shown to yield a homogenous population of erythroid progenitor cells fulfilling the strict host cell specificity and growth requirements of the erythrotropic parvovirus B19 [15]–[17]. The resulting CD36+ cells were generated with a defined combination of growth factors [7].

Parvovirus B19 comprising three major genotypes [18] belongs to the *Parvoviridae* family, genus *Erythrovirus* [17] and replicates selectively in erythroid progenitor cells at BFU-E and CFU-E stages [13], [19]. For this restriction, both *in vitro* investigations and clinical studies of this virus have been greatly hampered by the unavailability of fully permissive cell cultures. The *ex vivo*-derived CD36+ cell cultures [16] could thereby be of significant academic and practical utility via propagation of this clinically important virus, which hitherto has been done at best in primary cultures and semipermissive cell lines [11], [13], [20]–[29]. By using the same distinct growth factor composition [7], [16], we show that the methodology for obtaining erythroid stem cells can be markedly simplified, as performed without mobilization or CD34+ cell preselection, directly from ordinary samples of peripheral blood mononuclear cells (PBMC).

## Results

### Cell Culture

**Figure 1:**
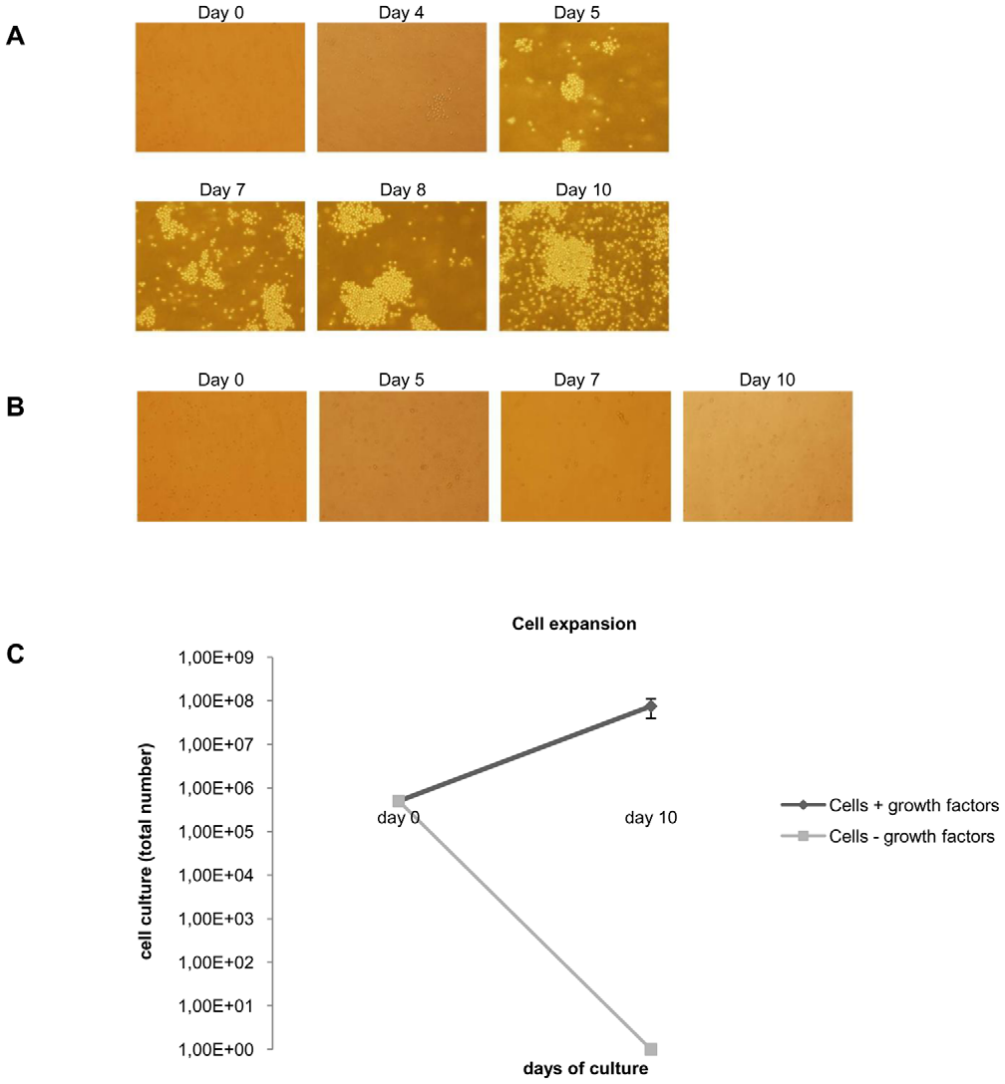
Phenotypic changes during cell culture from peripheral blood. (A) PBMC were isolated and cultured with growth factors favouring erythroid expansion and observed with an optical inverted microscope (Olympus IX71). Photographs were taken at 10× magnification with a Hamamatsu C8484-05G digital camera. (B) PBMC cultured as in panel A, but without the growth factors. (C) Numbers of cells cultured with or without the growth factors. Error bars indicate standard deviation.

5×105 freshly separated PBMC from the blood samples of staff members or the buffy coats of healthy donors were cultured in the presence of growth factors favouring the differentiation of pluripotent stem cells toward the erythroid lineage [7], [16]. In parallel, an equal amount of cells was cultured without the growth factors. On days 4 or 5 the first signs of cell clustering were seen in the cultures exposed to the growth factors (Fig. 1A). At this stage, the cells were split 1∶5 and cultured for 5 additional days. From the initial 5×105 cells their number increased to (5–10)×107 on day 10 (Fig. 1C); the overall increment was (1–2)×102-fold. In phenotype, the cells with the growth factors showed a progressive increase both in size and number, and particularly in extent of clustering (Fig. 1A) reaching a maximum by day 10 (Fig. 1C). In the absence of growth factors, apoptotic bodies and cell debris abounded, until on day 10 no living cells were seen (Figs. 1B–C).

### Flow Cytometry

**Figure 2:**
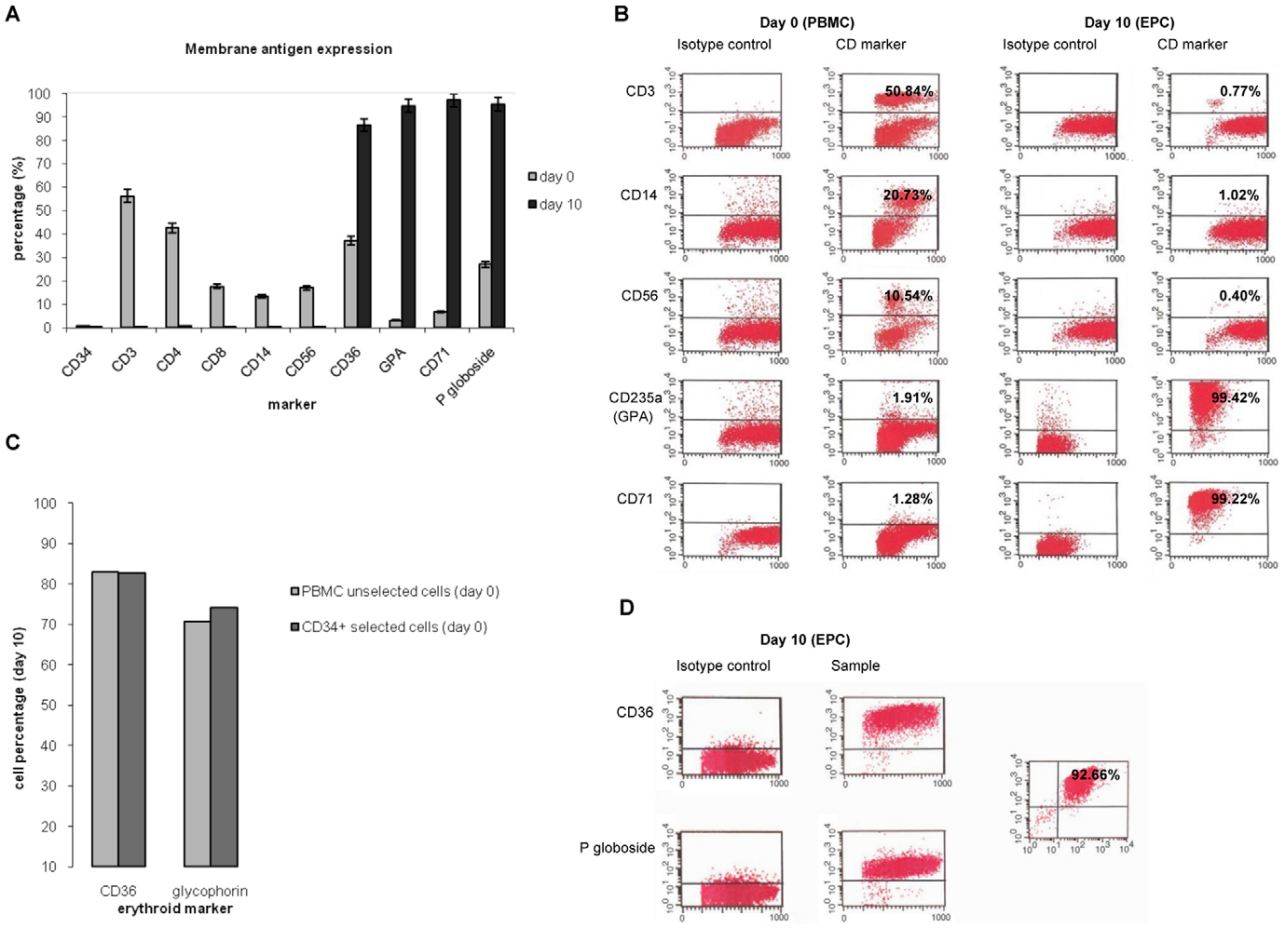
Flow cytometry analysis during culture with growth factors. Expression of cellular membrane antigen markers on day 0 and after 10 days of culture in presence of growth factors. FITC or PE labelled primary monoclonal antibodies for CD markers were used for cell staining. For each monoclonal antibody the correspondent anti-isotype antibody was used in parallel to test the specificity of the staining. Polyclonal rabbit antibody and antirabbit FITC were used to detect globoside P. (A) The histogram represents the percentages of positive cells to each marker analyzed; average of 5 experiments. Bars indicate percentage error. (B) Flow cytometry patterns of main lymphocyte, monocyte and erythroid markers on day 0 and on cultured cells. Representative experiment. Images were corrected for color uniformity by AdobePhotoshop software. (C) Comparison between unselected PBMC and CD34^+^-selected cells as culture source. Expression of CD36 and glycophorin on day 10 of culture. Representative experiment. (D) Expression of CD36 and globoside P on day 10. Representative experiment.

PBMC were analysed by flow cytometry immediately following separation (day 0) and after 10 days of culture with the growth factors. The cells were analysed for the main lymphocytic, monocytic and erythroid marker expression patterns. During this time a profound increase in erythroid cells was observed, with a decrease in lymphocytes and monocytes (Figs. 2A–B). On day 0, only <0,25% of the cells were CD34+. Specifically, the day 0 cell population comprised primarily lymphocytes and monocytes, >50% CD3+ (CD4+ or CD8+), ∼10% CD56+, and ∼20% CD14+ (Fig. 2B). All these values approached 0 on day 10. On the other hand, the erythroid progenitor cells that were barely detectable on day 0 increased to ∼95–100% by day 10, as indicated by CD71 and glycophorin expression patterns (Figs. 2A–B). Importantly, on day 10, CD36 positivity reached with some donors ∼100% of the cells. On day 0 this marker showed variable expression, in coexpression with the monocyte marker CD14 [30]. Of note, some of our donors presented with mononuclear CD36+ CD14− cells, as recently identified [31]. As with CD36, on day 0, also the proportion of globoside P-expressing cells showed variability among the donors, with a highest value of >20% (Fig. 2B), while on day 10 of culture nearly every cell co-expressed this B19 virus receptor along with CD36 (Fig. 2D).

### Comparison of Erythroid Progenitor Cells Generated from Unselected PBMC and Selected CD34+ Cells

In order to determine which cell type(s) represented the growth factor target(s), we followed in parallel cultures from unselected PBMC and from pure populations of preselected CD34+ cells. The cultures were analyzed by flow cytometry on days 0 and 10. Differentiation of the CD34+ cells into erythroid progenitors fully comparable with those from the unselected PBMC could be verified. Indeed, as opposed to the very low fraction of CD34+ cells in the PBMC on day 0 (Figs. 2A–B), the percentages of glycophorin+ and CD36+ erythroid progenitors were ≥70% and ≥80%, respectively, from the cultures of unselected PBMC and selected CD34+ cells (Fig. 2C).

### B19 Virus Permissivity of the Generated Erythroid Progenitor Cells

The erythroid progenitor cells expanded from peripheral blood were analysed for their functional competence by a virological approach. As the cells harboured both globoside P antigen and CD36 (Fig. 2D), they were considered suitable for a B19 parvovirus *in vitro* infection assay.

#### *In vitro* infection

Both of the procedures performed [16], [32] turned out comparable in all the downstream analyses. Furthermore, we observed no difference in any of the B19 infection parameters between the cells obtained from B19 seropositive and seronegative donors.

#### Nucleic acid analyses

**Figure 3:**
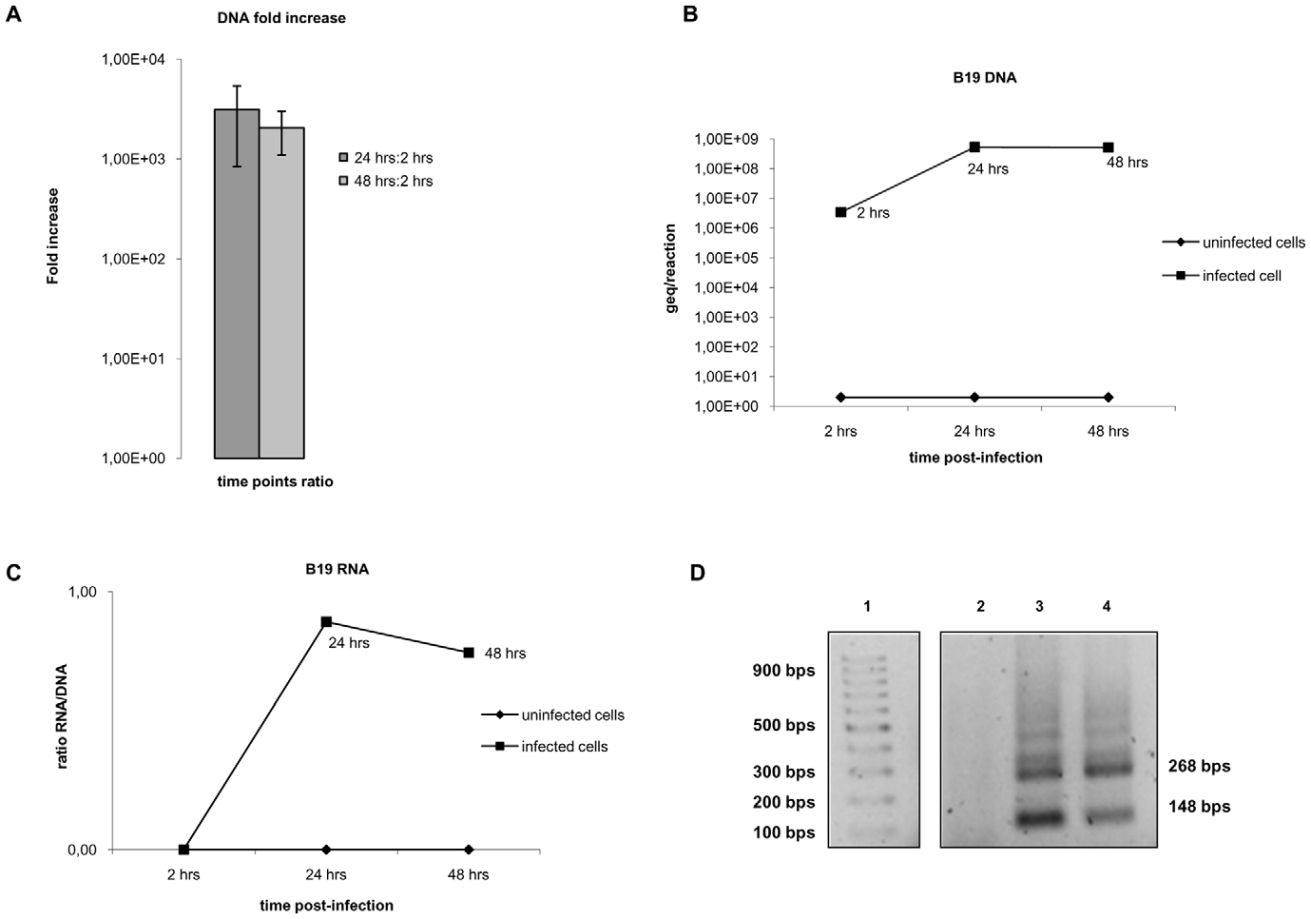
Cellular B19 virus DNA and RNA levels during in vitro infection. DNA and RNA were extracted following B19 in vitro infection of the obtained EPC. Real-time PCR and RT-PCR were performed. (A) B19 DNA fold increase at 24–48 hrs *versus* 2 hrs post-infection. Average of 3 experiments. Error bars indicate standard deviation. (B) Viral DNA pattern in cells harvested after in vitro infection. Absolute quantification was determined following qPCR of the extracts at different time-points. Representative experiment. (C) Relative amount of B19 RNA in cells harvested after infection. The obtained values take into account the qPCR results of the DNA template and the RNA in both presence and absence of retrotranscriptase. Representative experiment. (D) Agarose gel analysis of real-time RT-PCR amplicons. Lane 1: molecular weight markers. Spliced RNA in cells at 2 hrs (lane 2), 24 hrs (lane 3) and 48 hrs (lane 4) post-infection.

DNA and RNA were extracted from the infected and uninfected cells at 2, 24 and 48 hrs, and real-time PCR and RT-PCR were performed. The contiguous primers annealing to the common exon of the B19 genome were used for both DNA and RNA detection, the latter after DNase treatment. DNA was quantified by interpolation on a standard curve obtained with serial dilutions of plasmid DNA containing the coding region of the B19 genome. An overall increment of 3 logs of the DNA copy numbers was observed at 24–48 hrs post infection (Fig. 3A). Our assessment of the total B19 mRNA signal (Fig. 3C) took into account both the amount of DNA amplified by PCR (Fig. 3B) in absolute numbers and the extent of background DNA signal obtained by RT-PCR in the absence of reverse transcriptase. In RNA detection, the spliced VP transcripts, corresponding to the bands of 148 and 268 bp, were seen in agarose gel electrophoresis (Fig. 3D) following amplification with the non-contiguous primers [33].

#### Protein expression

**Figure 4:**
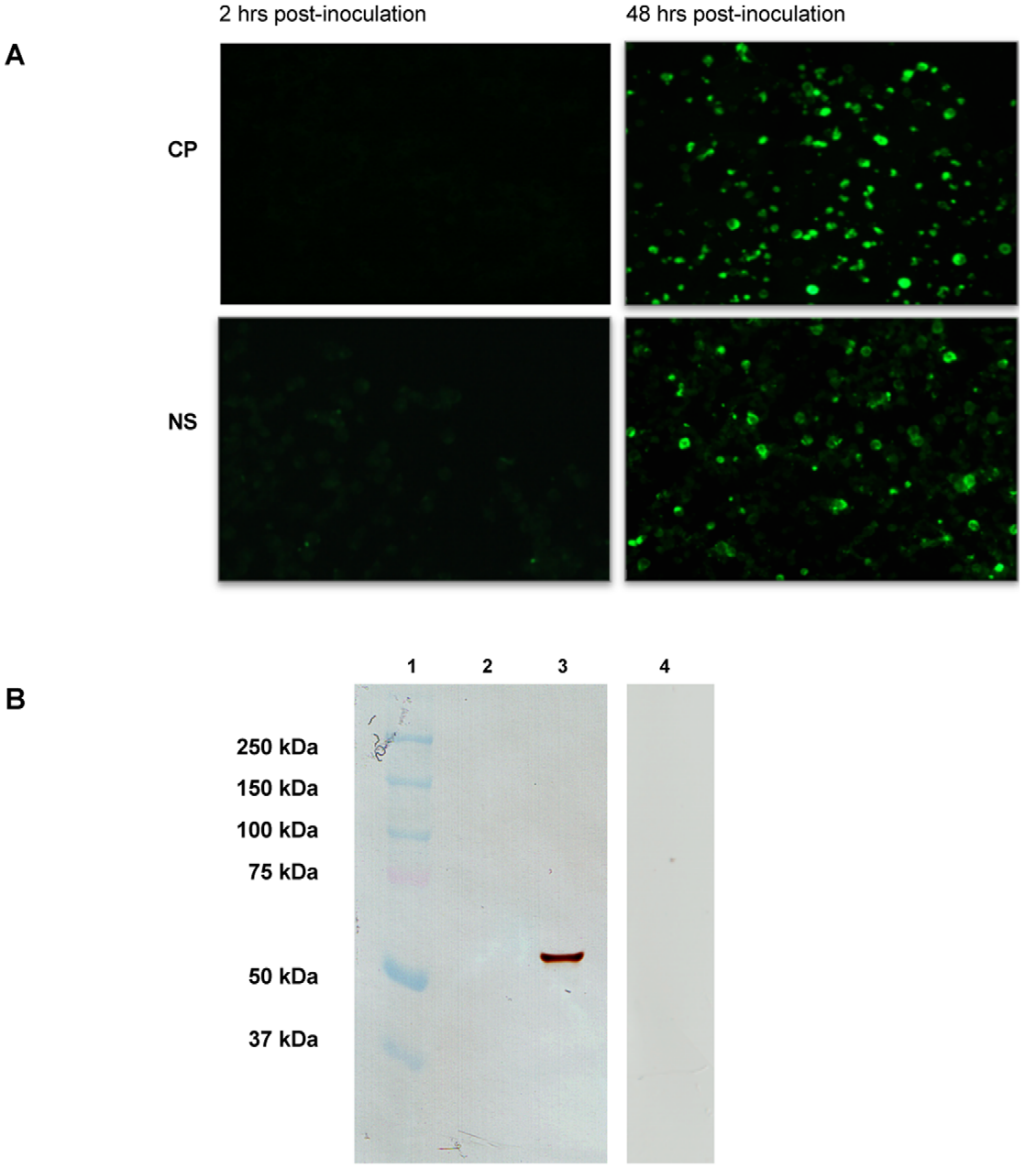
Permissivity of EPC for parvovirus B19 virus infection. Viral protein expression. (A) Immunofluorescence staining for viral capsid (VP) and nonstructural (NS1) proteins of cells infected on day 10 of culture, and fixed at 2 or 48 hrs post-infection. Monoclonal mouse antibody and human antibody were used to detect VP1-VP2 and NS1 respectively, followed by anti-mouse or anti- human FITC antibodies. The cells were observed with a Zeiss Axioplan 2 UV microscope. Photographs taken at 20× magnification. (B) Western blotting of lysates of uninfected (lane 2) or infected (lane 3) cells labeled with the monoclonal B19 capsid protein (VP) antibody. Infected cells labeled with isotype control antibody (lane 4). Lane 1: molecular weight markers.

The erythroid progenitor cells were analyzed for both structural (VP2) and nonstructural (NS1) proteins of the B19 virus, and in both native and denaturing conditions (Fig. 4). Immunofluorescence staining was performed on the infected and uninfected cells fixed at 2 and 48 hrs. At 48 hrs post-infection >50% of the cells were positive for VP2 and ∼50% for NS1, by contrast to 0% at 2 hrs post infection (Fig. 4A). Correspondingly, in Western blotting a strong VP2 band (58 kDa) was obtained from the cells lysed at 48 hrs post-infection, as opposed to none from the negative control cells (Fig. 4B).

### Extent of Increase of Erythroid Progenitor Cells

**Figure 5:**
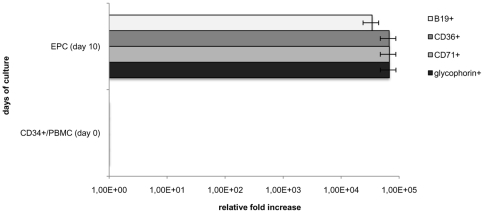
Expansion of EPC cultured from unselected PBMC. Grey bars: fold-increase of cells expressing CD36, CD71 and glycophorin markers on day 10 of culture. The values are based on the flow cytometry analysis of Figure 2 and the cell counts of Figure 1C and Table 1. White bar: fold-increase of B19 virus permissive EPC; percentage of cells expressing capsid proteins at 48 hrs post-infection (Fig. 4A). Bars represent error percentage.

Considering the proportion of hemopoietic CD34+ stem cells initially present in the unselected PBMC (Figs. 2A–B) and the expansion of overall cell number during culture (Fig. 1C), we first calculated the relative fold increase of the cells expressing the erythroid progenitor markers (CD34 vs. CD36 and CD71 along with glycophorin) (Fig. 5: grey bars; Table 1: rows 1, 2, 3). Moreover, the immunofluorescence results, in which >50% of the cells infected on day 10 expressed the B19 antigens, were taken into account together with the aforementioned variables, to calculate the fold increase of B19 permissive cells. As shown in Figure 5 (white bar) and Table 1 (row 4), (i) the overall cell count increased 100–200 fold; and concomitantly with this (ii) the proportion of EPC multiplied from <0.5% to >50%. I.e. it can be concluded that, during the *in vitro* culture our B19 permissive erythroid cells expanded >10 000-fold.

**Table 1:** Expansion of EPC directly from peripheral blood.

*Erythroid markers*	Fraction (%) day 10	Total relative fold increase
*EPC glycophorin+*	99.42	6.78E+04
*EPC CD71+*	99.22	6.77E+04
*EPC CD36+*	99	6.75E+04
*EPC B19+*	50	3.41E+04

Rows 1–3: Fold increase of cells expressing erythroid markers CD36, CD71 and glycophorin on day 10 of culture; the values are based on CD34^+^ fraction (%) on day 0 (0.22%) and on the expansion factor of 150, expressed as an average fold increase throughout the culture (Fig. 1C). The first column indicates the values obtained during the flow cytometry analysis of the three markers (Fig. 2B).

Row 4: Fold increase of B19 virus permissive cells (row 4); the values are based on CD34^+^ fraction (%) on day 0 (0.22%) and on the expansion factor of 150, expressed as an average fold increase throughout the culture (Fig. 1C). The first column indicates the percentage of cells expressing capsid proteins (Fig. 4A).

EPC represents erythroid progenitor cells.

## Discussion

For *in vitro* erythropoiesis many different approaches have been taken, utilizing pluripotent stem cells from a variety of sources [1]–[4], [6], [9], [34]. We used unfractionated PBMC obtained from ∼1 ml of peripheral blood of ordinary blood donors in the absence of bone marrow stem cell mobilization or of *ex vivo* priming of any other type, and selectively expanded the contained stem cells by *in vitro* culture in the presence of a defined combination of growth factors favouring erythropoietic commitment and expansion [7]. Indeed, our approach did not include any cell preselection, neither positive, for CD34+ [8]–[11], nor negative, against CD3-CD14- [13], nor adherence depletion [1], [3], and not even phased culturing [6], [12], [14] except for a single passage. Our straightforward approach yielded the desired population of erythroid progenitor cells comparable in purity and yield to the previously introduced approach including mobilization and preselection. Our comparison between selected CD34+ stem cells and unselected PBMC verified qualitatively and quantitatively similar yields of EPC. By this robust approach the initially diverse PBMC evolved *in vitro*, with natural selection toward a notably uniform population of colony-forming cells phenotypically characteristic of erythroid progenitors [6], [8], [12], [14], [35]. Of the cells cultured for 10 days nearly 100% expressed the marker combination of erythroid progenitors, CD36, CD71 and glycophorin, and were devoid of the lymphocyte, monocyte or NK markers CD4, CD8, CD3, CD14, CD56.

The aforementioned markers define best the immunophenotype of erythroid progenitor cells, as shown in a large number of studies [6], [8], [12], [14], [35]–[37]. In those studies glycophorin and the transferrin receptor CD71 have been identified as the reference molecules of erythroblasts. Although a well-established marker for erythroid progenitors [2], [16], CD36 on its own is far from cell type specific, as it occurs within the PBMC in monocytes, macrophages and even platelets, and participates in a variety of functions [30], [38]. However, we consider it appropriate to include CD36 as an indicator of erythropoietic commitment in concert with glycophorin and CD71 [10], [16], which were almost absent at the beginning yet occurred in nearly every cell at the end of our expansive culturing.

The expanded EPC were verified to be functionally competent by permissivity to B19 parvovirus infection. The B19 *in vitro* infection procedures employed here have been previously used in the documentation of functionality of mobilized, preselected (CD34+) cells [16], [32]. Our EPC obtained bypassing those steps were similarly susceptible to B19 virus infection, in terms of DNA replication (increase of 3 logs), mRNA transcription and protein production (both NS and capsid). Neither the presence of antiviral antibodies nor virus-specific T cells [39] in the donor blood affected the virological outcome.

Previous studies have addressed the role of accessory cells in establishing ideal conditions for the erythropoietic proliferation and commitment [1], [3], [6], [36], [40], [41]. In a comparative study with different cell lineages [36], cellular diversity has turned out to be essential compared with selected pluripotent cells. Our main finding, obtained with the novel procedure, is in line with this. The identification of the factors accounting for the differentiation within this condensed *in vitro* population is an interesting subject of further investigation.

Our procedure for EPC generation is gratifyingly robust. This straightforward technique can be foreseen to facilitate B19 virus basic research both in its molecular and cellular aspects, as well as to simplify the measurement of B19 virus neutralizing immunity [39], [42]–[46]. The latter studies have been hampered greatly by the absence of facile cell culture methodology for this clinically important virus [17], [47].

## Materials and Methods

### Ethics Statement

All the samples used during this study were obtained following written informed consent from the donors. This study was approved by the Ethical committee of the Helsinki University Central Hospital.

### Blood Samples and Cells

Peripheral blood samples were obtained into Vacutainer tubes from nonsymptomatic staff members. Leukocyte-enriched buffy coats of healthy blood donors were obtained from the Finnish Red Cross Blood Transfusion Service, Helsinki, Finland. From these preparations peripheral blood mononuclear cells (PBMC) were isolated by Ficoll-Paque centrifugation and two PBS washes [39], [42].

### Cell Culture

From the PBMC, erythroid progenitor cells (EPC) were generated via culture in an expansion medium containing erythropoietic growth factors [7], [16]. Briefly, 5×105 PBMC were cultured in MEM (HyClone, Logan, UT) supplemented with the serum substitute BIT 9500 (StemCell Technology, Vancouver, BC, Canada), diluted 1∶5 for a final concentration of 10 mg/ml bovine serum albumin, 10 µg/ml rhu insulin, and 200 µg/ml iron-saturated human transferrin, enriched with 900 ng/ml ferrous sulfate (Sigma, St. Louis, MO, USA), 90 ng/ml ferric nitrate (Sigma), 1 µM hydrocortisone (Sigma), 3 IU/ml rhu erythropoietin (Janssen-Cilag, Espoo, Finland), 5 ng/ml rhu IL-3 (R&D Systems, Minneapolis, MN, USA), and 100 ng/ml rhu stem cell factor (Peprotech, London, UK). The cells were maintained at 37°C in 5% CO2 and observed daily with an inverted microscope (Olympus IX71, Center Valley, PA, USA) for phenotypic changes. For control, PBMC of the same donors were grown in MEM either without the aforementioned substances or with only BIT (BSA, insulin, transferrin).

Upon observation of the initial small clusters on day 5±1, the cultures were split (1∶5) into their respective media.

For comparison, from some of the blood samples, CD34+ cells were first isolated by CD34 Microbead Kits (Miltenyi Biotech, Bergish Gladbach, Germany) containing magnetic beads conjugated to CD34 antibody, and were then cultured as described.

### Flow Cytometry

5×105 of each cell population were analysed both before culture (day 0) and on day 10 with FACScan (Becton Dickinson, Franklin Lakes, NJ, USA). The cells were washed thrice (in PBS containing 2% FCS) and treated for 30 min at 4°C with FITC-labelled monoclonal antibodies for CD34, CD45, CD71, CD1a, CD3, and with phycoerythrin (PE)-labelled monoclonal antibodies for CD36, CD14, CD56, and glycophorin (BD Biosciences, San Jose, CA, USA); and with a mixture of anti-CD4-PE and anti-CD8-FITC (DakoCytomation, Glostrup, Denmark). Anti-isotype antibodies (BD Biosciences) were used in parallel, for specificity control. Globoside P antigen, the B19 virus cell surface receptor [19], was examined by polyclonal rabbit antibodies (Matreya, Pleasant Gap, PA, USA) followed by anti-rabbit FITC (DakoCytomation). All the stained cells were washed thrice with, and resuspended in, PBS (200 µl).

### Parvovirus B19 Permissivity Study

#### Serology

All the PBMC donors' sera were studied for the presence of B19 virus IgG and IgM antibodies as described [43].

#### *In vitro* infection

Cell cultures of day 10 were infected at 103 genome equivalents per cell with B19 virus from a high-titer viremic, B19-seronegative donor plasma [48]. Two different in vitro infection procedures were used in parallel [16], [32]. In one, 2×104 cells in 10 µl volumes on a 96-well plate were treated with 10 µl of diluted virus for 2 hrs at 4°C, washed and maintained at a final concentration of 2×104/100 µl, at 37°C. In the other, 2×106 cells in 500 µl volumes on a 24-well plate were treated with the virus at 4°C, under agitation, washed and maintained at a final concentration of 2×105/ml, at 37°C. For control, mock-infected cultures, without addition of virus, were maintained in parallel with the infected ones. Aliquots of the test and control cultures were harvested at 2, 24 and 48 hrs post infection for DNA, RNA and protein analyses.

#### Nucleic acid analyses

DNA and RNA were extracted from the infected and uninfected cells at various times post infection, as described [33], [49]. Briefly, 105 cells were lysed with Proteinase K (100 µg/ml; Fermentas Finland, Helsinki, Finland) and 1% SDS in 100 mM NaCl, 10 mM EDTA, 10 mM Tris-Cl pH 7.5 for 1 h at 55°C, and the nucleic acids were extracted by phenol-chloroform followed by ethanol/Na-acetate precipitation. RNA was selectively extracted with ToTally RNA purification kit (Ambion, Austin, TX, USA) and treated with DNase TurboDNAfree (Ambion). A constant amount of both extracts, corresponding to ∼104 cells per reaction, was amplified, in parallel for viral DNA and mRNA, by the Light Cycler (Roche Applied Sciences, Basel, Switzerland) by using Quantitect SYBRgreen PCR and Quantitect SYBRgreen RT-PCR Kits (Qiagen, Hilden, Germany), respectively. The contiguous primers, corresponding to the common central exon of the viral genome and its thermal profile have been described [33], [49]. The absolute quantity of viral DNA was determined by interpolation using a standard curve constructed with serial dilutions (108–100 geq) of a plasmid containing the viral coding region (GenBank AY504945). Standards were included in each assay and linearity of the curve was confirmed. The RT-PCR was performed in the presence (RT+) and absence (RT-) of reverse transcriptase with the primer pair used for DNA amplification. The actual signal due to mRNA was obtained taking into account the Ct values [RT+] and [RT−], and the Ct corresponding to the DNA template. The relative increase at the various time points was then calculated. For additional evidence of RNA specificity of detection, non-contiguous primers (HR6) for spliced transcripts [33] were used. Specificity of the real-time PCR and RT-PCR results was confirmed by melting curve analysis and agarose gel electrophoresis of the amplicons for the contiguous and the non contiguous primer pairs, respectively. For extraction control, PCR for beta-actin DNA was performed in parallel [50].

#### Protein analysis

Cell-associated B19 proteins were visualized by immunofluorescence microscopy. At 2 hrs and 48 hrs post-infection 105 cells were spotted on glass slides (Biomerieux, Marcy l'Etoile, France), air-dried and fixed in methanol-acetone (1∶1) for 10 minutes at −20°C. Monoclonal mouse antibody for both VP1 and VP2 capsid proteins (clone 521-5D; Chemicon Millipore, Billerica, MA, USA) or monoclonal human antibody for NS1 protein [51] (a generous gift from Susanne Modrow) were visualized by anti-mouse or anti-human IgG-FITC (DakoCytomation), respectively. After washing and embedding, fluorescence was observed and photographed with a Zeiss Axioplan 2 (Carl Zeiss, Jena, Germany) UV microscope.

Western Blotting of lysates from ∼3×104 infected or uninfected cells was performed as described [42] with reducing 7.5%DS-PAGE gels, by using a monoclonal B19 virus capsid antibody (NovoCastra Laboratories, Wetzlar, Germany) followed by anti-mouse IgG-HRP (DakoCytomation). The corresponding isotype control antibody (R&D Systems) was applied for specificity control.
